# Supplementary material for: Splice-Junction-Based Mapping of Alternative Isoforms in the Human Proteome
Source: Cell Rep. Author manuscript; Available in PMC 2020 Jan 15. (PMC6961840; doi:10.1016/j.celrep.2019.11.026)
Supplement: 2 [file NIHMS1546469-supplement-2.zip › DF1/PXD010154/Lung/Lung_17_MTA1_RDYVYFENSSSNPYLIR.pdf]

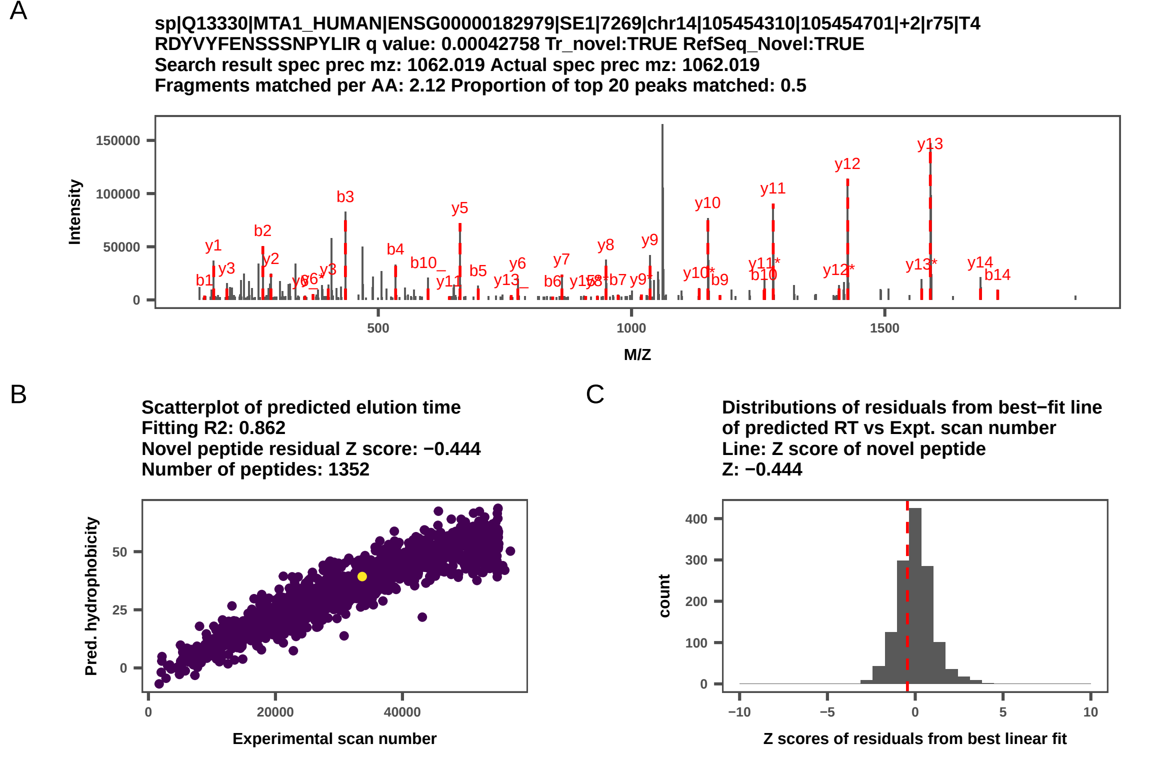
A
sp|Q13330|MTA1_HUMAN|ENSG00000182979|SE1|7269|chr14|105454310|105454701|+2|r75|T4
RDYVYFENSSSNPYLIR q value: 0.00042758 Tr_novel:TRUE RefSeq_Novel:TRUE
Search result spec prec mz: 1062.019 Actual spec prec mz: 1062.019
Fragments matched per AA: 2.12 Proportion of top 20 peaks matched: 0.5
Intensity
150000
100000
50000
0
M/Z
500
1000
1500
y1
b1
y3
b2
y2
b3
b4
b10_
y5
b5
y6
y7
y8
y9
y10
y10*
b9
y11
y11*
b10
y12
y12*
y13
y13*
y14
b14
B
Scatterplot of predicted elution time
Fitting R2: 0.862
Novel peptide residual Z score: −0.444
Number of peptides: 1352
Pred. hydrophobicity
50
25
0
0
20000
40000
Experimental scan number
C
Distributions of residuals from best−fit line
of predicted RT vs Expt. scan number
Line: Z score of novel peptide
Z: −0.444
count
400
300
200
100
0
−10
−5
0
5
10
Z scores of residuals from best linear fit
